# Mechanisms of immunotherapy in cutaneous squamous cell carcinoma in the tumor microenvironment

**DOI:** 10.3389/fimmu.2025.1660272

**Published:** 2025-11-17

**Authors:** Qinyi Dong, Zijian Zhang, Siying Li, Lili Liang

**Affiliations:** 1 The Third Clinical College, Shanxi University of Chinese Medicine, Taiyuan, China; 2 Department of Plastic and Aesthetic Surgery, Shanxi Provincial People’s Hospital Affiliated to Shanxi Medical University, Taiyuan, China

**Keywords:** cutaneous squamous cell carcinoma, tumor microenvironment, immunotherapy, immunosuppression, immune checkpoint inhibitors

## Abstract

Cutaneous squamous cell carcinoma (cSCC) is a common cutaneous malignant tumor, with its development and progression closely linked to immune dysregulation within the tumor microenvironment (TME). This review highlights cSCC-specific TME features—such as UV-induced mutational burden and the immunosuppressive effects observed in transplant recipients—and systematically outlines the composition and functional roles of tumor cells, immune cells (Tregs, MDSCs, TAMs), and stromal cells (CAFs) within the TME. The immunosuppressive mechanisms mediated by these cellular components are clarified, particularly through pathways including PD-L1/PD-1 and TGF-β/Smad. Building on this foundation, the potential clinical value of immune checkpoint inhibitors (cemiplimab, pembrolizumab) in treating advanced cSCC is summarized based on data from relevant clinical trials. Additionally, the impact of gender differences on cSCC incidence and therapeutic outcomes is discussed. This review is distinguished from general tumor immunotherapy reviews by offering dedicated references for cSCC precision immunotherapy. In addition, priority is emphasized for future investigations into combination therapy regimens and the development of personalized tumor vaccines.

## 1 Preamble

### 1.1 Clinical epidemiological characteristics of cSCC

Squamous cell carcinoma of the skin (cSCC) accounts for 20%–30% of cutaneous malignant tumors, and its incidence has been increasing steadily over the years. Although the fatality rate of squamous cell carcinoma of the skin is significantly lower than that of melanoma—with a 5-year survival rate of approximately 60% for melanoma and around 30%–40% for advanced cSCC (1)—it remains higher than that of basal cell carcinoma, which has a 5-year survival rate exceeding 95% and rarely metastasizes. Therefore, effective treatment of cSCC is crucial for reducing mortality associated with skin cancer. The primary etiological factor of cSCC is exposure to ultraviolet (UV) radiation. Prolonged UV exposure can cause DNA damage in skin cells, leading to gene mutations—such as those in TP53—which in turn promote tumor development (2).

### 1.2 cSCC morbidity and prognostic gender differences

A higher incidence of cSCC has been observed in males, with incidence rates approximately 2–3 times greater than those in females. This disparity may be attributed to prolonged outdoor exposure to ultraviolet radiation and lower awareness of skin protection among men (3). Regarding tumor progression, male patients with cSCC tend to exhibit greater tumor aggressiveness and higher rates of lymph node metastasis. These differences are underpinned by immunological and biological mechanisms rather than solely by environmental exposure. In a cohort of 1,178 primary cSCC cases, males demonstrated significantly higher risks of high-grade tumors, thick-walled lesions, lymph node involvement, and long-term recurrence compared to females (P < 0.001). Analysis of the tumor microenvironment showed that the densities of intralesional CD8^+^/CD4^+^ T cells and M1 macrophages in males were 40%–60% lower than those in females, indicating that insufficient immune infiltration may contribute to a more aggressive tumor phenotype (4). Currently, no dedicated studies have investigated gender differences in the efficacy of immune checkpoint inhibitors (ICI) for cSCC, and thus, it remains unclear whether objective response rates differ between males and females. It is recommended that future clinical trials systematically incorporate sex-based analyses to better elucidate this issue.

### 1.3 Association between the tumor microenvironment and immunotherapy in cSCC

The TME of cSCC constitutes a complex network of cellular and molecular components, with its immunosuppressive state playing a pivotal role in immune surveillance evasion and the emergence of therapeutic resistance. In recent years, significant advances have been achieved with immune checkpoint inhibitors in the treatment of advanced cSCC. However, a proportion of patients still experience either primary or acquired resistance, which is closely linked to the accumulation of MDSCs and functional dysregulation of Tregs within the TME (5, 6). Therefore, a comprehensive investigation into the composition and regulatory mechanisms of the TME in cSCC is essential for optimizing immunotherapeutic strategies and enhancing clinical outcomes.

## 2 The role of the tumor microenvironment in cSCC

### 2.1 Composition of the tumor microenvironment

**Figure 1:**
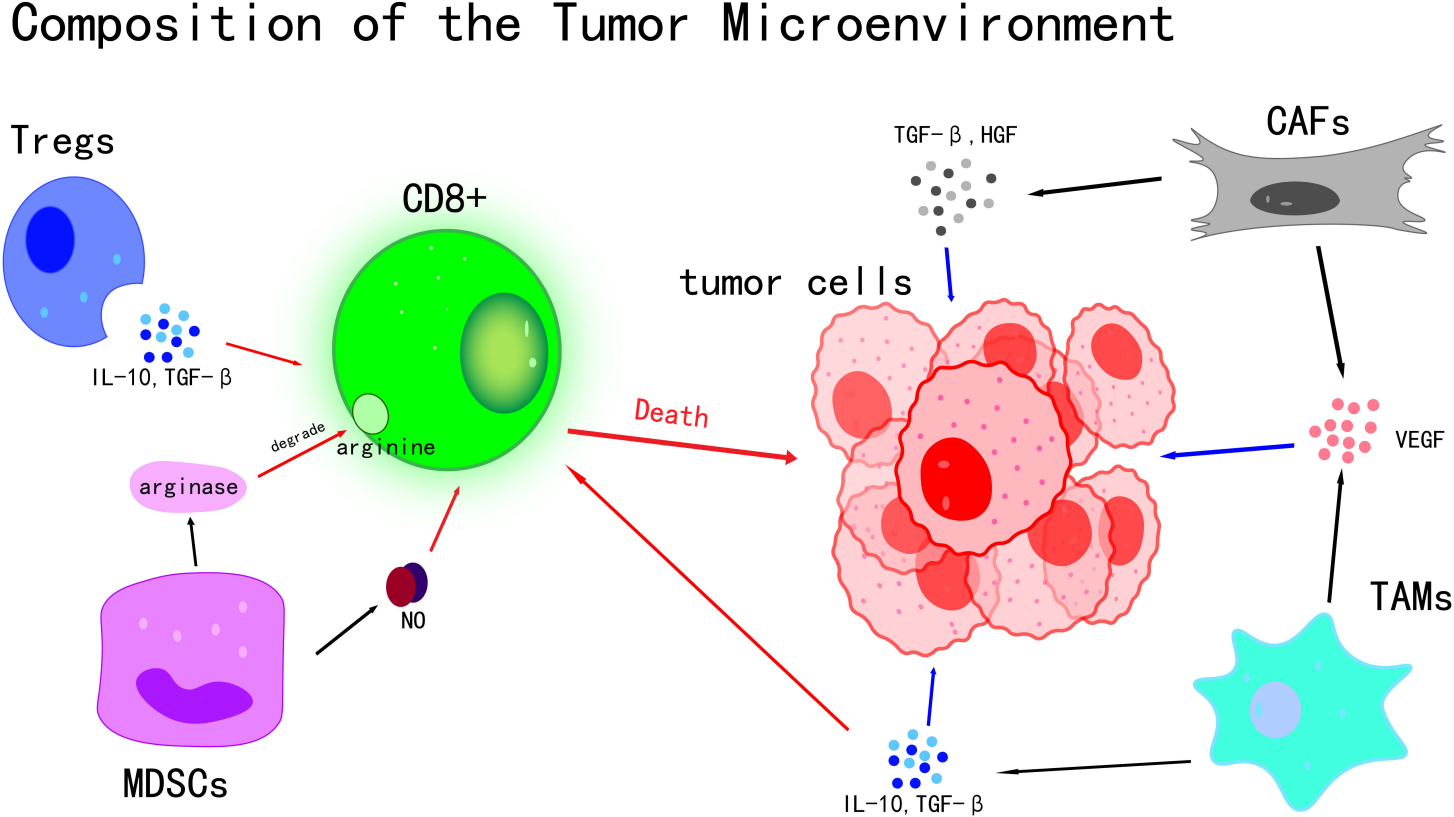
Composition of the tumor microenvironment.

The tumor microenvironment (TME) is a complex and dynamic system that plays a pivotal role in tumor initiation, progression, and metastasis (7). As the central component of the TME, Tumor Cells exhibit a range of distinctive biological characteristics that allow them to bypass normal physiological regulatory mechanisms, leading to uncontrolled proliferation, invasion, and metastatic potential (8). Rather than existing in isolation, Tumor Cells actively secrete various Cytokines and Chemokines, which significantly influence the behavior of surrounding cells. Through these interactions, a microenvironment favorable to tumor growth and Immune Evasion is established (9) (**Figure 1**). A high Mutational Burden induced by UV has been observed in cSCC Tumor Cells, with an average mutation frequency of approximately 50 mutations per Megabase, which is markedly higher than that found in most other Solid Tumors. This elevated mutational load can result in the production of more Tumor Antigens, such as mutant TP53 protein. However, immune evasion is also facilitated through mechanisms including the downregulation of Major Histocompatibility Complex (MHC) class I molecules and the upregulation of PD-L1 (10).The link between mutational burden and immune evasion is especially evident in areas such as the face and scalp, which are chronically exposed to UV radiation, leading to a higher propensity for local tumor progression.

immune cells exhibit highly complex roles within the TME, characterized by dual biological functions (11). On one hand, they can recognize and attack Tumor Cells, thereby suppressing tumor growth (11, 12); on the other hand, under certain conditions, immune cells may be subverted by Tumor Cells, ultimately facilitating tumor progression (12). Among the various subsets of immune cells, T cells are recognized as pivotal players in the anti-tumor immune response. Effector T cells, especially CD8+ T cells, function as precise “killers” capable of specifically identifying antigens presented on the surface of Tumor Cells. Upon activation, they initiate a cascade of cytotoxic responses that directly eliminate Tumor Cells (13).In contrast, regulatory T cells (Tregs) play an opposing role in the Tumor Microenvironment. The activity of effector T cells is suppressed by Tregs through the secretion of various inhibitory Cytokines, including IL-10 and TGF-β (14). These inhibitory Cytokines disrupt the proliferation, activation, and cytotoxic functions of effector T cells (15, 16), thereby impairing the body’s Immune Surveillance and antitumor response, and facilitating the Immune Evasion of Tumor Cells.

Myeloid-derived suppressor cells (MDSCs) are a population of myeloid-origin cells with potent immunosuppressive properties (17). Their immunosuppressive effects are mediated through multiple mechanisms, with the secretion of inhibitory molecules such as arginase and nitric oxide (NO) being one of the primary pathways (18).Arginine, an amino acid essential for T cell proliferation and activation, is degraded by arginase, resulting in impaired T cell function (19, 20). Nitric oxide further suppresses T cell proliferation and activity by disrupting normal physiological functions through mechanisms such as oxidative stress (18, 21), thereby weakening the body’s anti-tumor immune response.

High plasticity has also been observed in macrophages within the TME, with their phenotype and function dynamically altered in response to various signaling molecules present in the microenvironment (22). In the Tumor Microenvironment, these macrophages can differentiate into pro-tumor M2-type macrophages, also known as tumor-associated macrophages (TAMs).Various Cytokines, such as IL-10 (23) and TGF-β (24), are secreted by TAMs. Acting synergistically, IL-10 and TGF-β establish an immunosuppressive microenvironment that inhibits anti-tumor immune responses. This environment not only suppresses T cell activity but also promotes the proliferation and invasion of Tumor Cells, thereby directly contributing to tumor progression (25, 26).Additionally, growth factors secreted by TAMs, including vascular endothelial growth factor (VEGF) (27), stimulate tumor angiogenesis (28). This neovascularization supplies Tumor Cells with ample nutrients and oxygen, thereby supporting sustained tumor growth and metastasis (29).

Stromal cells are also essential components of the TME, primarily comprising cancer-associated fibroblasts (CAFs) and endothelial cells. Multiple roles have been attributed to CAFs in tumor initiation and progression. By secreting a variety of Cytokines, such as TGF-β and HGF, along with growth factors like VEGF, significant influences are exerted on the biological behavior of Tumor Cells (30). These factors have been shown to promote the proliferation of Tumor Cells, facilitating their rapid growth and expansion. Additionally, they enhance the migratory capacity of Tumor Cells, thereby contributing to tumor invasion and metastasis (30, 31).

Endothelial cells are primarily involved in tumor angiogenesis (32).In the early stages of tumor development, a series of angiogenic factors, such as VEGF, are secreted by Tumor Cells, which attract endothelial cells to migrate toward the tumor site and promote their proliferation and lumen formation (33). The role of VEGF in the Tumor Microenvironment extends beyond angiogenesis. It also modulates intercellular interactions within the Tumor Microenvironment, including the regulation of pericyte proliferation and migration, as well as the mediation of interactions between tumor-associated macrophages and carcinoma cells. These interactions contribute to PDL-1-mediated immunosuppression and Nrf2-driven epithelial-mesenchymal transition (EMT) (34). The newly formed tumor vasculature not only ensures an adequate blood supply to meet the high metabolic demands of Tumor Cells for nutrients and oxygen, but also facilitates their metastasis by providing a direct route for dissemination through the bloodstream to distant sites, where secondary metastatic lesions can form.

The extracellular matrix (ECM), as a crucial structural and functional component of the TME, has been shown to significantly influence the invasion and metastasis of Tumor Cells through alterations in its composition and architecture (35). It is primarily composed of collagen, fibronectin, and laminin (36). In terms of compositional changes, increased collagen deposition has been observed in the Tumor Microenvironment, particularly with upregulated expression of collagen types I and III (37). This accumulation contributes to tissue stiffening, thereby facilitating the invasive behavior of Tumor Cells (35). Elevated levels of hyaluronic acid (HA) have also been detected in various tumors, especially low-molecular-weight HA oligosaccharides, which interact with CD44 receptors to activate pro-tumor signaling pathways and enhance cell migration and invasion (37).Alterations in the expression and deposition of fibronectin have been observed, which affect cell adhesion and migration (35). Structurally, increased cross-linking of collagen fibers results in greater stiffness and rigidity of the extracellular matrix (ECM). This enhanced stiffness activates intracellular mechanotransduction pathways, including the integrin and focal adhesion kinase (FAK) signaling cascades, thereby promoting the invasion of Tumor Cells. In the Tumor Microenvironment, collagen fibers become more orderly aligned, forming channels that facilitate the migration of Tumor Cells (37).

Within the TME, Cytokines and signaling molecules act as messengers, establishing a complex and efficient communication network between Tumor Cells and surrounding cells, and playing a pivotal regulatory role (38).Cytokines are small-molecule proteins secreted by various types of cells, including interleukins (IL), interferons (IFN), and tumor necrosis factors (TNF). They exert dual functions within the TME, both promoting tumor growth, metastasis, and Immune Evasion, and activating immune cells to suppress tumor progression (39). For instance, Cytokines such as IL-6 and TGF-β, secreted by Tumor Cells, have been shown to inhibit the activity of immune cells, thereby facilitating Immune Evasion. This is achieved through the suppression of immune cells proliferation, differentiation, and Cytokines production, ultimately weakening the body’s Immune Surveillance and cytotoxic response against tumors. As a result, Tumor Cells are able to survive and proliferate within a relatively “safe” immune environment, enabling the realization of Immune Evasion (39).immune cells (such as tumor-associated macrophages TAMs) have been shown to secrete Cytokines (such as IL-1β and IL-18), which promote the proliferation and metastasis of Tumor Cells (38).

Moreover, metabolic byproducts within the TME have been found to play a significant role in tumor progression. Glutamine metabolism is critically involved in the function of Tumor Cells (40). A high dependency on glutamine is exhibited by Tumor Cells, as it is utilized through the glutamine fermentation pathway to generate energy necessary for their rapid growth and proliferation. Glutamine serves as a nitrogen source for the biosynthesis of amino acids and nucleotides, and also as a carbon source to replenish the tricarboxylic acid (TCA) cycle, thereby sustaining accelerated cellular proliferation (41).Meanwhile, the acid-base balance of the TME has been shown to be altered by Tumor Cells through metabolic byproducts such as lactate (42). The accumulation of lactate results in the acidification of the TME, creating an acidic microenvironment that suppresses immune cells by diminishing their activity and function. This immunosuppressive environment further promotes the immune evasion of Tumor Cells and creates more favorable conditions for their invasion and metastasis (42, 43).

Pyroptosis, a form of programmed cell death, contributes to the remodeling of the TME by promoting the release of pro-inflammatory cytokines and sustaining chronic inflammation, thereby affecting processes such as Immune Evasion and angiogenesis. However, its dual role in both cancer progression and treatment-related complications—such as cytokine release syndrome—poses significant challenges for its therapeutic application in oncology (44).

Key cellular components and their interactions within the Tumor Microenvironment are illustrated. The death of Tumor Cells is induced by CD8+ T cells through direct cytotoxic mechanisms. The activity of CD8+ T cells is suppressed by Tregs via the secretion of IL-10 and TGF-β. T cell function is inhibited by MDSCs through the production of arginase and nitric oxide (NO). CAFs and TAMs contribute to tumor growth and angiogenesis by releasing TGF-β, HGF, and VEGF, thereby fostering an immunosuppressive microenvironment. Collectively, these cellular interactions shape tumor progression and influence the response to therapy.

### 2.2 Immunoregulatory role of the tumor microenvironment

The TME has been identified as a central target for elucidating the mechanisms underlying tumor initiation and progression, as well as for addressing therapeutic challenges. As a dynamic and complex multicellular ecosystem, the TME encompasses not only Tumor Cells but also immune cells, stromal cells, and components of the extracellular matrix. It plays a pivotal role in tumor Immune Evasion and progression through tightly regulated cellular and molecular pathways. Clarifying the immunoregulatory mechanisms of the TME, especially the activation patterns of immunosuppressive pathways and potential strategies for their modulation, is of great clinical significance for the development of effective anti-tumor therapies.

#### 2.2.1 Core pathways of immunosuppressive mechanisms

Multiple core pathways have been identified in the TME of cSCC that contribute to the formation of an immunosuppressive barrier. Among them, the PD-L1/PD-1 and TGF-β/Smad pathways represent key molecular mechanisms driving Immune Evasion. These pathways impair anti-tumor immune responses by respectively inhibiting the activation signals and proliferative capacity of effector T cells.

The PD-L1/PD-1 pathway, a principal target in current immunotherapy, displays distinct cell-type specificity during its activation. In the TME of cSCC, high levels of PD-L1 expression have been observed in both Tumor Cells and TAMs (45). Upon binding of PD-L1 to PD-1 on effector T cells, immunosuppressive signaling cascades such as the PI3K/Akt pathway are activated within the T cells (46).Activation of this signaling pathway has been shown to directly suppress the proliferative capacity of T cells and reduce the secretion of cytotoxic molecules such as perforin and granzymes. As a result, effector T cells are unable to effectively recognize and eliminate Tumor Cells, thereby promoting tumor Immune Evasion (47). Clinical studies have demonstrated that immune checkpoint inhibitors targeting this pathway (e.g., Cosibelimab) can restore the antitumor activity of T cells by blocking the interaction between PD-L1 and PD-1, leading to significant survival benefits in patients with cSCC (48).

The TGF-β/Smad pathway primarily relies on TGF-β secreted by Tregs and cancer-associated CAFs. Upon binding of TGF-β to its receptor on the surface of effector T cells, a downstream Smad signaling cascade is initiated. This involves the phosphorylation of Smad2/3 transcription factors, which then form a heterotrimeric complex with Smad4. The resulting complex translocates into the nucleus, where it directly regulates the expression of target genes. The primary effect of this process is the downregulation of the co-stimulatory molecule CD28 on the surface of T cells, accompanied by a decrease in IL-2 secretion. CD28, a critical “second signal” molecule required for T cell activation, when downregulated, results in impaired activation of T cells (49, 50). Concurrently, IL-2, a key Cytokines essential for T cell proliferation, when secreted in reduced amounts, directly suppresses the clonal expansion of T cells. Sustained activation of this pathway maintains effector T cells in the TME in a functionally suppressed state, thereby hindering the development of an effective antitumor immune response.

It is important to note that immunosuppression within the TME is not driven by a single pathway, but rather arises from the synergistic effects of multiple signaling cascades, including the PD-L1/PD-1 and TGF-β/Smad pathways. For example, the activation of the Smad pathway by Tregs through TGF-β secretion is accompanied by the release of IL-10, which further amplifies their immunosuppressive effects (51). In parallel, the overexpression of PD-L1 on Tumor Cells enables its interaction with PD-L1 on the surface of TAMs, forming a “dual barrier” that intensifies the suppression of T cell activity (52). This synergistic immunosuppressive network involving multiple pathways is a key factor contributing to the limited effectiveness of conventional immunotherapy.

#### 2.2.2 Association between TME cellular and molecular pathways and tumor progression

Cellular and molecular pathways within the TME have been shown to not only regulate immune responses but also directly contribute to malignant behaviors such as invasion and migration of Tumor Cells. Notably, the EMT pathway mediated by CAFs and the STAT3 pathway associated with MDSCs represent critical hubs that link immunosuppression to tumor progression. By modulating the phenotype of Tumor Cells and the function of immune cells, these pathways collectively drive the malignant advancement of cSCC.

The EMT pathway mediated by CAFs is recognized as a central mechanism facilitating the invasion and metastasis of cSCC. As the predominant type of stromal cells within the TME, CAFs secrete TGF-β, which activates the TGF-β/Smad signaling cascade in Tumor Cells, thereby inducing EMT (53). Specifically, TGF-β secreted by CAFs binds to the TGF-β receptor complex (TGF-β R1/R2) on the surface of Tumor Cells, initiating conformational changes that activate downstream Smad2/3 proteins (54). Once phosphorylated, Smad2/3 forms a transcriptional complex with Smad4, which translocates into the nucleus. This complex simultaneously downregulates the epithelial marker E-cadherin—whose loss disrupts intercellular adhesion among Tumor Cells and reduces cell cohesion (55)—and upregulates mesenchymal markers such as N-cadherin and vimentin, thereby enhancing the motility and stromal invasiveness of Tumor Cells (56).Multiple clinical studies have demonstrated a significant association between the activation level of this pathway and the depth of invasion in cSCC, underscoring its critical role in tumor progression (57, 58).

The STAT3 pathway in MDSCs has been shown to promote both Immune Evasion and tumor proliferation through a dual mechanism involving immunosuppression and metabolic deprivation. MDSCs, a key immunosuppressive cell population within the TME, rely heavily on the sustained activation of signal transduction and the STAT3 pathway for their functional maintenance (59). During the initiation and progression of cSCC, UV irradiation serves as a major contributing factor, markedly increasing IL-6 secretion within the TME (60). IL-6 binds to its receptor on the surface of MDSCs, leading to the activation of Janus kinase (JAK), which in turn phosphorylates STAT3 (61). Once activated, STAT3 translocates into the nucleus, where it directly regulates the expression of arginase-1 (Arg-1) and inducible nitric oxide synthase (iNOS) (62).Arg-1 depletes arginine, an amino acid essential for T cell activation in the TME, thereby impairing T cell proliferation due to “metabolic starvation” (63). Meanwhile, iNOS produces large amounts of nitric oxide (NO), which damages T cell DNA and suppresses their cytotoxic function, resulting in a dual immunosuppressive effect of “metabolic deprivation + toxic injury” (64). In animal studies, treatment with STAT3 inhibitors (such as WP1066) has been shown to significantly reduce the accumulation of MDSCs in murine tumor models, while restoring T cell proliferation and cytotoxic activity (65), supporting the potential of this pathway as a therapeutic target.

Beyond these two core pathways, the Cytokines network within the TME also contributes to tumor progression through a mechanism of cross-regulation. For example, activation of the MDSCs STAT3 pathway by the pro-inflammatory Cytokines IL-6 has been identified as a key signaling event. In addition, IL-6 promotes STAT3 activation in Tumor Cells, thereby enhancing their resistance to apoptosis and increasing their proliferation rate (66). Meanwhile, the immunosuppressive Cytokines TGF-β not only inhibits T cell function but also upregulates the expression of matrix metalloproteinases (MMPs) on the surface of Tumor Cells, enhancing their capacity to degrade the extracellular matrix and further facilitating invasive metastasis (67). This intercellular signaling crosstalk between immune and tumor cells establishes a self-perpetuating malignant cycle within the TME, thereby accelerating tumor progression and metastasis.

#### 2.2.3 cSCC TME immunosuppressive mechanisms and corresponding regulatory strategies

From a clinical translational perspective, targeting a single pathway has often proven insufficient to overcome the immunosuppressive network of the TME, making combination therapy strategies a growing focus of research. For instance, the co-administration of anti-PD-1 antibodies with STAT3 inhibitors has been shown to simultaneously disrupt PD-L1/PD-1-mediated immunosuppression and the functional maintenance pathways of MDSCs, thereby significantly enhancing T cell activation efficiency (68). Similarly, the combination of anti-TGF-β and anti-PD-L1 antibodies has been demonstrated to suppress tumor EMT progression while improving the efficacy of immune checkpoint inhibitors (69) (**Table 1**). Looking ahead, molecular subtyping based on key pathways within the cSCC TME is expected to guide the development of individualized combination therapy regimens, representing a promising direction for enhancing the therapeutic outcomes of cSCC.

**Table 1:** cSCC TME Immunosuppressive Mechanisms and Corresponding Regulatory Strategies.

Immunosuppressive mechanism	Key molecules/pathways	Regulatory strategies	Effects
Tregs suppression of effector T cells	IL-10, TGF-β	Anti-IL-10 antibodies, Anti-TGF-β antibodies	Significantly reduce the immunosuppressive activity of Tregs, increase the proportion of effector T cells in the TME (relative increase +25%), and enhance the intensity of anti-tumor immune responses
MDSCs deplete arginine/produce NO	Arginase-1, iNOS, STAT3	STAT3 inhibitors (e.g., WP1066), Arginine supplementation	Reduce the accumulation of MDSCs in the TME (relative decrease -40%), reverse the “metabolic starvation” state of T cells, and restore their proliferation and cytotoxic functions
Tumor cell PD-L1/PD-1 binding	PD-L1, PD-1	Anti-PD-1/PD-L1 antibodies (e.g., Pembrolizumab)	Block PD-L1/PD-1-mediated immunosuppressive signals, significantly increase the objective response rate (ORR) to 47%-52.9%
CAFs promoting tumor EMT	TGF-β/Smad	Anti-TGF-β antibodies	Downregulate the expression of mesenchymal markers such as N-cadherin and vimentin, reduce tumor invasion depth (relative decrease -48%), and inhibit tumor metastatic potential

## 3 Justification for the priority of immunotherapy in cSCC

### 3.1 Comparison with surgical treatment

Surgical resection, owing to its advantage of directly eliminating lesions, is considered the first-line curative approach for early-stage cSCC (characterized by localized tumors without lymph node or distant metastasis), achieving a 5-year cure rate of over 90% (70). However, its primary limitation lies in spatial dependence. In advanced-stage (III–IV) cSCC, where tumors invade major blood vessels in the head and neck (e.g., the carotid artery), cranial nerves, or are accompanied by multiple metastases to organs such as the lungs or liver, complete surgical removal becomes challenging, with a curative rate of less than 10%. Moreover, surgery may result in serious complications, including major intraoperative bleeding and postoperative swallowing dysfunction, and could even accelerate tumor dissemination—surgical trauma may activate platelet-derived growth factors within the TME, thereby promoting Tumor Cells migration (71, 72). Immunotherapy has addressed this limitation by “remodeling the TME immune balance.” In a phase II clinical trials (NCT02760498) evaluating the first-line agent cemiplimab for advanced cSCC, 59 patients with metastatic cSCC were enrolled. An objective response rate (ORR) of 47% was observed, and the median overall survival (OS) was not reached; at a median follow-up of 7.9 months, 82% of responders maintained their response (73). In comparison, conventional treatment—surgery combined with postoperative chemotherapy—has been associated with poorer outcomes. Analysis of the SEER database revealed that patients with advanced cSCC, particularly those with regional lymph node metastases or locally advanced disease, who underwent surgery and adjuvant radiotherapy, had a median OS often below 12 months, indicating a significant disparity (74). More critically, “tumor downstaging conversion” can be achieved through immunotherapy by inhibiting the PD-L1/PD-1 pathway within the TME and reducing the accumulation of MDSCs (75). Clinical data indicate that, following neoadjuvant immunoradiotherapy, 90% of patients with locally advanced head and neck squamous cell carcinoma (HNSCC) experienced clinical-to-pathological downstaging, and 67% achieved a pathological complete response (pCR) (76).

### 3.2 Comparison with radiotherapy

Radiotherapy induces DNA damage in Tumor Cells via localized ionizing radiation and is applicable to locally advanced cSCC (e.g., tumors infiltrating the deep dermis without distant metastasis), with a five-year local control rate of approximately 65%. However, its limitations include a restricted treatment scope and damage to the TME. On one hand, radiotherapy is ineffective against distant metastases, with a distant metastasis rate of 15%–20% observed in patients with advanced cSCC receiving this treatment (77). On the other hand, radiotherapy induces local skin fibrosis, characterized by a 30% increase in collagen fiber deposition, and leads to depletion of immune cells within the TME. Following radiotherapy, PD-1 expression on T cells increases by 40%, and the proportion of M2-type TAMs rises by 20%, thereby further aggravating immunosuppression (78).

The characteristic of “systemic immune activation” in immunotherapy effectively addresses this limitation. First, a significant reduction in the rate of distant metastasis in advanced cSCC has been observed with immunotherapy, primarily through the activation of circulating effector T cells that recognize and eliminate metastatic lesions (79).Secondly, Immunotherapy also contributes to the restoration of the radiation-damaged TME. Tumor antigens released by radiotherapy, such as mutant TP53 protein, can be presented by dendritic cells. Concurrently, immune checkpoint inhibitors (e.g., pembrolizumab) can block the PD-L1/PD-1 signaling pathway, thereby preventing effector T cell exhaustion and promoting a synergistic effect between radiotherapy-induced antigen release and immune activation (80). A combined trial demonstrated that the use of radiotherapy in conjunction with pembrolizumab in patients with locally advanced cSCC resulted in a higher response rate, with a complete response (CR) rate reaching up to 50%, and was well tolerated (79). Furthermore, the combination of radiotherapy and immunotherapy has been shown to enhance the production of antitumor Cytokines such as IFN-γ, while reducing the infiltration of immunosuppressive cells, including M2-type TAMs. This mechanism has been validated in multiple Solid Tumors, such as non-small cell lung cancer (80).In addition, immunotherapy has been shown to reduce adverse reactions associated with radiotherapy. Multiple studies have demonstrated that the safety profile of immunotherapy combined with radiotherapy is generally manageable, with immune-related adverse events (such as skin reactions) typically being mild to moderate. For instance, in the KEYNOTE-629 trial, the incidence of grade ≥3 treatment-related adverse events with pembrolizumab monotherapy was reported to be 5.7% (81).

### 3.3 Comparison with targeted therapy

Targeted therapy for cSCC primarily relies on EGFR inhibitors (such as cetuximab), which act by blocking EGFR-mediated Tumor Cells proliferation signaling. However, three major limitations have been identified: rapid development of resistance, limited applicability, and lack of improvement in the TME. First, the median time to resistance with cetuximab is only 6–9 months (82), and this resistance is associated with changes in the TME—following resistance, increased secretion of HGF by CAFs within the TME activates the MET bypass pathway, thereby counteracting the inhibitory effects of EGFR (83). Second, the efficacy of cetuximab is restricted to patients with high EGFR expression (IHC score ≥2+), yet only 60% of cSCC patients exhibit such expression levels (84), with an objective response rate (ORR) of merely 20%–30% (85). Finally, targeted therapy does not modulate the immunosuppressive state of the TME, and even after resistance develops, the proportion of Tregs within the TME remains above 15%, thereby failing to elicit a sustained anti-tumor immune response (86).

Immunotherapy has demonstrated advantages through its “broad-spectrum efficacy” and “TME remodeling.” In terms of applicability, immune checkpoint inhibitors have shown effectiveness in both PD-L1-positive (TPS ≥ 1%) and -negative patients (87, 88). Regarding the durability of response, multiple trials have reported that the median duration of response (DOR) has not been reached; for example, in the KEYNOTE-001 trial, the median DOR was 12.5 months, with some patients experiencing responses lasting over two years. A notably high proportion of patients in KEYNOTE-001 maintained a response for ≥1 year (87), which is believed to be associated with the induction of immune memory. Following treatment, the proportion of memory T cells (CD45RO+) in the TME increases, enabling long-term surveillance of tumor recurrence (89).Regarding adverse events, the incidence of grade 3 rash with targeted therapy is approximately 6.1%–13% (90), and diarrhea is also relatively common. In contrast, the incidence of grade 3 adverse events associated with immunotherapy—such as thyroid dysfunction and rash—ranges from about 9.5% to 29.6%, most of which can be effectively managed with hormonal intervention (87, 91, 92).More importantly, the TME can be fundamentally improved through immunotherapy. Treatment with anti-PD-1 has been shown to reduce the expression of MDSCs functional markers, such as arginase-1, while enhancing the infiltration of effector T cells. For instance, preclinical studies have demonstrated that PD-1 blockade decreases the presence of immunosuppressive cells within the TME (93), enabling some patients to regain responsiveness to treatment—an effect on TME modulation that cannot be achieved by targeted therapies.

## 4 Application of immunotherapy in cSCC

### 4.1 Immune checkpoint inhibitors

The emergence of immune checkpoint inhibitors (ICIs) in recent years has revolutionized tumor therapy, offering renewed hope to countless cancer patients. By precisely blocking the signaling pathways of immune checkpoint molecules, ICIs effectively restore the antitumor activity of effector T cells and markedly enhance the immune response (94), thereby ushering in a new era of cancer immunotherapy.

Immune checkpoint molecules serve as critical regulatory components of the immune system, functioning under normal physiological conditions to prevent excessive immune activation that could harm healthy tissues. However, Tumor Cells have evolved to exploit these molecules, converting them into mechanisms that suppress T cell activity. This enables them to evade immune surveillance and establish conditions conducive to their growth and metastasis. Currently, among the various immune checkpoint molecules, CTLA-4, PD-1, and PD-L1 have been the most extensively studied and hold significant clinical relevance. CTLA-4 plays a critical role during the early activation phase of T cells. By binding to members of the B7 family (95, 96), it modulates T cell activation or suppression. Inhibitors of CTLA-4, such as ipilimumab (97), function by precisely blocking the interaction between CTLA-4 and its ligands, thereby lifting the inhibitory signal. This facilitates enhanced activation and proliferation of T cells (96), effectively acting as a potent “vanguard” for the ensuing anti-tumor immune response.

In contrast, PD-1 is primarily involved during the effector phase of T cell responses. Upon binding of PD-1 to its ligand PD-L1, an inhibitory signal is transmitted to T cells, resulting in the suppression of their activity and impairing their ability to effectively recognize and eliminate Tumor Cells (98). By precisely blocking this signaling pathway, PD-1/PD-L1 inhibitors—such as pembrolizumab (99) and cemiplimab (100)—have been shown to successfully restore the antitumor function of T cells, enabling them to regain activity and mount effective attacks against Tumor Cells (101).

immune checkpoint inhibitors have demonstrated impressive therapeutic efficacy across a range of tumors, standing out like brilliant pearls in the vast landscape of cancer treatment.

#### 4.1.1 Key clinical trials results

cSCC (CSCC) is the second most common type of skin cancer after basal cell carcinoma. While most CSCC cases can be cured surgically, recurrence occurs in approximately 15% to 28% of patients following excision. For those with advanced CSCC who are ineligible for curative surgery or radiotherapy, treatment options remain limited and the prognosis is generally poor. In recent years, immune checkpoint inhibitors (ICI) have offered new therapeutic possibilities for advanced CSCC. Extensive efforts have been made by research teams to investigate the role of CPI in cSCC treatment through systematic reviews, meta-analyses, and cohort studies. These reviews have consistently demonstrated that CPI therapy yields favorable clinical outcomes in advanced cSCC, with manageable toxicity profiles (102–104) (**Table 2**).

**Table 2:** Key clinical trials Results.

Trial drug	Trial phase	Sample size	ORR	DCR	Median OS	Median PFS	Incidence of grade 3 or higher adverse events	Reference
Cemiplimab	Phase I/II	26	50%	65%	Not reached	Not reached	Not reported	Migden et al., 2018 (73)
Cemiplimab	Phase II (mCSCC)	59	47%	61%	Not reached	18.4 months	9.80%	Migden et al., 2020 (144)
Cemiplimab	Phase II (laCSCC)	78	44%	62.80%	Not reached	Not reached	Not reported	Rischin et al., 2021 (145)
Cemiplimab	Pooled Analysis	392	52.90%	69.40%	Not reached	Not reached	27.12%	Mehta et al., 2021 (103)
Pembrolizumab	Phase II (KEYNOTE-629)	105	34.30%	52.40%	Not reached	6.9 months	5.70%	Grob et al., 2020 (81)
Pembrolizumab	Phase II (CARSKIN)	57	42%	Not reported	Not reached	6.7 months	Not reported	Maubec et al., 2020 (106)
Cemiplimab/Pembrolizumab	Real-world study	286	60%	Not reported	12 months: 78%	12 months: 65%	19% (Grade 2 or higher)	McLean et al., 2024 (104)
Immune checkpoint inhibitors	Real-world study	36	69.50%	Not reported	12 months: 76.7%	21.3 months	13.90%	Koch Hein et al., 2023 (109)
Immune checkpoint inhibitors	Meta-analysis	980	47.20%	64.40%	6 months: 80.6%, 12 months: 76.4%	6 months:59.3%, 12 months: 52.8%	20.20%	Zhang et al., 2023 (45)

Among them, cemiplimab (105) and pembrolizumab (106–108) have distinguished themselves with outstanding therapeutic efficacy. Demonstrated in clinical trials, their strong antitumor activity and relatively favorable safety profiles have garnered significant attention from the global medical community. Based on this robust clinical evidence, the U.S. Food and Drug Administration (FDA) approved cemiplimab for the treatment of advanced cSCC, marking a milestone that effectively addressed the lack of treatment options for advanced cSCC. Cemiplimab, a PD-1 inhibitor, achieved an overall response rate (ORR) of 47% in a phase I/II clinical trials for CSCC, making it the first PD-1 antibody approved by the FDA for the treatment of advanced CSCC (102).In addition, a pooled analysis demonstrated that the objective response rate (ORR) of Cemiplimab in treating advanced CSCC was 52.9%, with a disease control rate (DCR) of 69.4%. The median overall survival (OS) was not reached, suggesting a survival duration exceeding 6.3 months. Another pooled analysis of 392 patients showed that immune checkpoint inhibitors (including Cemiplimab) achieved an ORR of 42.43% and a DCR of 58.05%, with 92% of patients maintaining a response at the data cut-off (103). Pembrolizumab, another PD-1 inhibitor, was assessed in two phase II trials (KEYNOTE-629 and the CARSKIN trial), which reported an ORR ranging from 34.3% to 41% for advanced CSCC, with a median progression-free survival (PFS) of 6.7 to 6.9 months (102).

A systematic review and meta-analysis that included 13 studies and a total of 980 patients reported that immune checkpoint inhibitors achieved an ORR of 47.2% and a DCR of 64.4%. The 6-month and 12-month progression-free survival rates were 59.3% and 52.8%, respectively, while the 6-month and 12-month overall survival rates were 80.6% and 76.4%, respectively (102). A retrospective real-world cohort study conducted in Australia included 286 patients, reporting an objective response rate (ORR) of 60%, a 12-month overall survival rate of 78%, and a progression-free survival rate of 65% (104). In another single-center retrospective cohort study from Canada involving 36 patients, a partial response rate of 41.7% and a complete response rate of 27.8% were observed. The 1-year progression-free survival rate reached 58.1%, with a median progression-free survival of 21.3 months (109). Regarding drug safety, among the 392 patients included in the pooled analysis, grade 3 or higher adverse events were observed in only 27.12% of cases. In the Australian study, 19% of patients experienced grade 2 or higher immune-related adverse events, with no treatment-related deaths reported. In a Canadian study, grade 3–4 immune-related adverse events were reported in 13.9% of patients.

In the field of melanoma, nivolumab as a first-line treatment has offered new hope for therapy-naive melanoma patients (110). Significant survival benefits have been demonstrated in clinical studies, with notable extensions in overall survival and improvements in quality of life. In the treatment of non-small cell lung cancer, the superiority of PD-1/PD-L1 inhibitors in first-line therapy has been consistently supported by multiple studies. Compared with conventional chemotherapy, these inhibitors not only significantly increase survival rates but also improve the overall treatment experience, presenting a promising therapeutic alternative for patients with non-small cell lung cancer (111).

The objective response rate (ORR) of cemiplimab in patients with advanced cSCC has been reported at 47%–52.9%, which is slightly lower than that observed in other Solid Tumors, such as melanoma, where the ORR exceeds 50% in some studies. Nevertheless, given that a substantial proportion of cSCC patients are elderly (approximately 70% aged ≥65 years), have impaired immune function, and often present with chronic skin conditions (e.g., chronic eczema), this level of efficacy reflects meaningful clinical benefit. Furthermore, the incidence of grade ≥3 adverse events associated with immune checkpoint inhibitors in cSCC patients ranges from 19.0% to 27.12%, which is lower than the rates reported in melanoma (exceeding 30% in some studies), suggesting better overall tolerability in this population.

#### 4.1.2 Table of clinical impact and pharmacokinetic characteristics of common immunotherapies for cSCC

In the immunotherapy of advanced cSCC (cSCC), both cemiplimab (cemiplimab) and pembrolizumab (pembrolizumab) have shown notable clinical value, although differences exist in their pharmacokinetic profiles and predictive markers of efficacy (**Table 3**). Over the past five years, multiple trials have reported that the median overall survival (OS) for cemiplimab has not yet been reached, with the longest follow-up period being approximately 16.6 months. The 24-month OS rate has been estimated at around 62%, indicating a sustained survival benefit (112). Furthermore, the expression level of PD-L1 (TPS ≥1%) has been identified as a potential predictive biomarker for treatment response. Patients with PD-L1-positive tumors have demonstrated significantly higher objective response rates (ORR) compared to those with PD-L1-negative tumors. While data from other cancer types suggest ORRs of 58% versus 32%, a similar trend has been observed in cSCC studies, despite some variation in exact values (73, 113).In terms of pharmacokinetics, an increase in the clearance of pembrolizumab has been observed with increasing body weight—approximately an 8% rise in clearance for every 10 kg gain—necessitating weight-based dose adjustments (e.g., 2 mg/kg) to prevent subtherapeutic exposure that could compromise efficacy. In contrast, the half-life of cemiplimab remains stable (19–22 days) and is unaffected by age, sex, body weight, or mild hepatic or renal impairment, making a fixed-dose regimen (350 mg every 3 weeks) more suitable for standardization. Taken together, cemiplimab offers notable efficacy and convenient administration in advanced cSCC, with PD-L1 expression serving as a predictive biomarker of response, whereas pembrolizumab requires individualized dosing to optimize therapeutic outcomes.

**Table 3:** Clinical Impact and Pharmacokinetic Characteristics of Common Immunotherapies for cSCC.

Drug name	Target	ORR	Common adverse reactions	Half-life	Route of administration	Dosage
Cemiplimab	PD-1	47.0%-52.9%	Rash (35%), Diarrhea (20%)	About 20 days	Intravenous injection	350mg, once every 3 weeks
Pembrolizumab	PD-1	38.1%-47.0%	Thyroid dysfunction (22%), Fatigue (18%)	About 26 days	Intravenous injection	200mg, once every 3 weeks; or 400mg, once every 6 weeks
Atezolizumab	PD-L1	33.3%	Nausea (25%), Fatigue (21%)	About 27 days	Intravenous injection	1200mg, once every 3 weeks

However, the use of immune checkpoint inhibitors is not without risks, as some patients may develop immune-related adverse events (irAEs).These adverse reactions are primarily attributed to the “friendly fire” triggered by excessive activation of the immune system (114). Skin toxicity is among the most frequently observed side effects across multiple systems. Symptoms such as rash and pruritus are generally mild to moderate in severity, yet they can still cause considerable discomfort and negatively impact patients’ quality of life. Endocrine toxicity also warrants attention. Conditions like hypothyroidism and hypophysitis may insidiously disrupt hormonal balance, requiring regular monitoring and prompt medical intervention. Gastrointestinal toxicity remains a significant clinical concern. Diarrhea and colitis can severely compromise digestive function and, in severe cases, may pose life-threatening risks. In addition, hepatotoxicity may occur, resulting in abnormal liver function. This necessitates close monitoring of liver function parameters during the administration of immune checkpoint inhibitors to promptly detect and manage potential risks of liver injury.

In recent years, a series of encouraging breakthroughs have been reported in the field of immune checkpoint inhibitors, marking significant progress and continuously expanding the boundaries of our understanding of this therapeutic strategy. In the search for novel targets, emerging molecules such as LAG-3, TIM-3, and TIGIT have gained increasing attention, bringing new momentum to the development of immune checkpoint inhibitors (115–117).

Similarly, substantial progress has been made in the research of biomarkers. Biomarker studies have focused on PD-L1 expression levels, tumor mutational burden (TMB), and microsatellite instability (MSI). Assessment of PD-L1 expression serves as a critical reference for identifying patients who are likely to benefit from immune checkpoint inhibitors therapy (118). High TMB in Tumor Cells has been associated with the release of a greater number of Tumor Antigens, which can enhance immune activation and improve the therapeutic response to immune checkpoint inhibitors (119). This insight provides a novel perspective and valuable tool for predicting immunotherapy outcomes. Similarly, microsatellite instability (MSI) plays a key role in forecasting the efficacy of immune checkpoint inhibitors treatment (120).

### 4.2 Potential of bispecific antibody in the treatment of cSCC

Bispecific antibodies are a class of antibody drugs designed to simultaneously bind two distinct antigens. Their primary advantage is the targeted recruitment of immune cells, such as T cells, to the tumor site, thereby enhancing the local anti-tumor immune response while minimizing the systemic toxicity associated with off-target T cell activation often observed in conventional immunotherapy. In the treatment of cSCC, research has predominantly focused on EGFR/CD3 bispecific antibodys, with additional investigations exploring other targets such as PD-L1/CD3 and EpCAM/CD3. The following section presents a detailed analysis of EGFR/CD3 bispecific antibodies.

#### 4.2.1 Core mechanism of action

High expression of EGFR has been observed on the surface of cSCC Tumor Cells (121), while CD3 serves as a critical component of the TCR-CD3 complex on T cells, mediating the transmission of activation signals (122). The EGFR/CD3 bispecific antibody functions via a “dual-antigen bridging” mechanism. Specifically, its “tumor-targeting arm”—an anti-EGFR single-chain antibody fragment—selectively binds to EGFR on cSCC Tumor Cells, effectively anchoring T cells in close proximity to the Tumor Cells and minimizing off-target activation in non-tumor tissues (123). Concurrently, the “immune-activating arm”—an anti-CD3 single-chain antibody fragment—binds to the CD3ϵ chain on T cells, triggering intracellular signaling cascades such as the ZAP-70 and ERK1/2 pathways through cross-linking of the TCR-CD3 complex. This activation induces T cells to release cytotoxic molecules, including perforin and granzyme B, which directly eliminate the bound Tumor Cells (124). In addition, activated T cells secrete Cytokines such as IFN-γ and TNF-α, which further recruit additional effector T cells (e.g., CD8+ T cells) and natural killer (NK) cells into the Tumor Microenvironment, contributing to the establishment of long-lasting anti-tumor immune memory (124). Compared with conventional anti-EGFR monoclonal antibodies (such as cetuximab), EGFR/CD3 bispecific antibodies not only block EGFR-mediated tumor proliferation signaling but also actively recruit T cells to exert cytotoxic effects. This dual mechanism makes them particularly suitable for patients with cSCC characterized by immune-desert microenvironments, where T-cell infiltration is minimal (123).

#### 4.2.2 Preclinical evidence

Although no EGFR/CD3 bispecific antibodies targeting cSCC have yet entered the clinical trials stage, multiple preclinical studies have demonstrated their potent antitumor activity and favorable tolerability in various EGFR-overexpressing Solid Tumors models.

While specific investigations on cSCC remain limited, several studies employing cutaneous squamous cell carcinoma cell lines (e.g., A431) have confirmed the efficacy of EGFR/CD3 bispecific antibodies. In A431 cells, representing a model of cutaneous squamous cell carcinoma, these bispecific antibodies (such as the ATTACK format) have shown strong binding affinity and effectively inhibited downstream EGFR signaling pathways and cell proliferation (124).In the A431 xenograft model, significant inhibition of tumor growth was achieved by the EGFR/CD3 bispecific antibody through activation of T cell-mediated Tumor Cells killing (125).

Preclinical data from tumors with high EGFR expression, such as head and neck squamous cell carcinoma (HNSCC) and colorectal carcinoma, further support the potential application of the EGFR/CD3 bispecific antibody in cSCC. In HNSCC models (e.g., HNSCC cell lines), T cell-dependent lysis of Tumor Cells was induced by the EGFR/CD3 bispecific antibody (126). In colorectal carcinoma models, Probody-engineered EGFR/CD3 bispecific antibodies (e.g., CI107) demonstrated a marked reduction in toxicity while maintaining therapeutic efficacy (127).

#### 4.2.3 Current challenges

Despite encouraging preclinical findings, three key challenges must be addressed for the clinical translation of EGFR/CD3 bispecific antibodies in cSCC:

Cytokine release syndrome (CRS): Activation of T cells by bispecific antibodies may provoke an excessive immune response, resulting in the massive release of cytokines such as IL-6 and IL-1β, which can manifest as fever, hypotension, and organ dysfunction (128).In preclinical studies of EGFR/CD3bispecific antibody, the severity of CRS has been found to correlate with the extent of T-cell activation. However, this can be mitigated through engineering approaches, such as reducing CD3 binding affinity, which lowers Cytokines release while preserving antitumor efficacy (129).

Heterogeneous EGFR expression has been observed: approximately 10%–20% of cSCC patients exhibit low EGFR expression (IHC score 1+) or are negative in tumor tissues, potentially rendering them unresponsive to EGFR/CD3 bispecific antibodies (130). Higher EGFR expression levels have been reported in cSCC lesions exposed to greater UV radiation (e.g., face, scalp) compared to those on the trunk (mean IHC score 3+ *vs*. 2+), indicating the importance of biomarker-based patient selection (131).

off-target toxicity remains a concern, as normal epidermal keratinocytes also express low levels of EGFR (IHC score 1+). The bispecific antibody may bind to these normal skin cells and activate local T cells, potentially resulting in dermatologic toxicities such as rash, pruritus, and epidermal detachment (132). In preclinical mouse models, administration of high-dose EGFR/CD3 bispecific antibody (20 mg/kg) resulted in diffuse erythema on the skin, with histopathological analysis revealing lymphocytic infiltration in the epidermis. To mitigate binding to normal cells, optimization through antibody engineering is required (133).

### 4.3 Combination immunotherapy

At the forefront of tumor treatment, combination immunotherapy has garnered significant attention for its distinctive advantages. By strategically integrating multiple therapeutic approaches, it generates synergistic effects that enhance the efficacy of immunotherapy, offering novel perspectives and strategies for overcoming the challenges posed by tumors.

Chemotherapeutic agents play a pivotal role in combination immunotherapy. By inducing immunogenic cell death (ICD) of Tumor Cells, they facilitate the release of Tumor Antigens and activate the immune system, thereby augmenting the effectiveness of immune checkpoint inhibitors. The potential of this combined approach has been strongly supported by results from clinical trials (108). For instance, in patients with advanced cSCC (cSCC), the combination of pembrolizumab and chemotherapy significantly increased the objective response rate (ORR), leading to more substantial therapeutic benefits. This achievement highlights the synergistic benefits of combining chemotherapy with immune checkpoint inhibitors, offering new therapeutic avenues for the treatment of advanced cSCC.

The combination of targeted therapies with immune checkpoint inhibitors has also shown considerable therapeutic promise. Agents such as EGFR inhibitors (134) and BRAF inhibitors (135) effectively block critical signaling pathways in Tumor Cells, thereby suppressing their growth and survival. This suppression further enhances the antitumor efficacy of immune checkpoint inhibitors. In patients with EGFR-mutated cSCC, a treatment regimen combining pembrolizumab with cetuximab, an EGFR inhibitor, has demonstrated marked clinical efficacy. The overall safety of pembrolizumab in combination with cetuximab has been found to be manageable. The most common grade 3–4 adverse event reported is stomatitis, with no treatment-related deaths observed (136). These findings suggest that the combination therapy regimens are generally well tolerated in patients with advanced CSCC. The therapeutic mechanism of this combination involves the targeted agent impairing the defense mechanisms of Tumor Cells, thereby enabling immune checkpoint inhibitors to more effectively activate the immune system for a coordinated antitumor response. This synergistic interaction offers a more tailored treatment option for patients whose tumors carry specific genetic mutations.

Encouraging outcomes have also been achieved in clinical studies investigating the combination of radiotherapy with immune checkpoint inhibitors. High-energy radiation has been shown to destroy the DNA of Tumor Cells, thereby inducing immunogenic cell death (ICD) (137). During this process, a substantial amount of Tumor Antigens is released by Tumor Cells, which activates the immune system and enhances the anti-tumor efficacy of immune checkpoint inhibitors. According to clinical trials, the combination of radiotherapy and pembrolizumab significantly improves the objective response rate (ORR) in patients with locally advanced cSCC (79). These findings demonstrate that the synergistic use of radiotherapy and immune checkpoint inhibitors enables a multifaceted attack on tumors, thereby enhancing therapeutic outcomes and offering new hope for patients with locally advanced cSCC. Moreover, ICD induced by radiotherapy not only strengthens the local immune response against tumors but also converts “cold” tumors (characterized by a lack of lymphocytic infiltration) into “hot” tumors (responsive to immunotherapy), thereby improving the overall effectiveness of immunotherapeutic strategies (138). This combined therapeutic strategy, known as radioimmunotherapy, has demonstrated promising potential in the treatment of various cancers (139). In one study, a liposomal nanomedicine named C/J-LipoRGD was developed by co-encapsulating a biological enzyme and a BRD4 inhibitor, enabling tumor-targeted delivery and modulation of the TIME. This formulation improved the oxygenation status of the Tumor Microenvironment, reduced the expression of PD-L1, reversed T cell exhaustion, significantly suppressed tumor growth, and induced ICD, thereby activating a T cell-mediated antitumor immune response (140).

immune checkpoint inhibitors can also be combined with other innovative therapeutic approaches, such as oncolytic viruses (141) and radionuclide therapy (142). Oncolytic viruses represent a novel therapeutic strategy characterized by a unique antitumor mechanism. They selectively infect and lyse Tumor Cells, while simultaneously releasing a substantial amount of Tumor Antigens within the Tumor Microenvironment. This process further stimulates the immune system and triggers a new inflammatory response aimed at eliminating Tumor Cells (141). In parallel, radionuclide therapy employs the localized radiation effects of radioactive nuclides to precisely target Tumor Cells (143), while also activating immune responses and enhancing the efficacy of immune checkpoint inhibitors. These combination therapies offer more diversified treatment options and are anticipated to play an increasingly important role in future clinical applications.

In summary, combination immunotherapies markedly amplify antitumor immune responses through the synergistic action of multiple mechanisms, offering renewed hope for cancer patients. With ongoing research advancements and the integration of new technologies, combination immunotherapies are expected to be continually optimized and refined. Emerging therapeutic strategies will be developed, existing regimens will become more precise and efficient, and personalized treatment approaches will see broader implementation. These advancements are anticipated to further enhance patient survival rates and quality of life, driving cancer treatment toward greater precision, efficacy, and individualization.

## 5 Conclusion

A critical role has been established for the TME of cSCC in tumor initiation, progression, and immune evasion. The Tumor Microenvironment constitutes a complex ecological system comprising immune cells, stromal cells, Cytokines, and the extracellular matrix (ECM). Through intricate interactions, these components collectively modulate tumor growth, invasion, and metastasis. Comprehensive exploration of the immunoregulatory mechanisms within the Tumor Microenvironment—including the recruitment and activation of immune cells, the establishment of an immunosuppressive milieu, Immune Evasion by Tumor Cells, and the regulation of Cytokines networks—has laid a robust theoretical foundation for the development of innovative immunotherapeutic strategies.

Immunotherapy, particularly the use of immune checkpoint inhibitors and approaches targeting the immunosuppressive microenvironment, has shown marked efficacy in the treatment of cSCC, offering promising prospects for enhancing patient survival and quality of life. However, immunotherapy may also lead to immune-related adverse events, such as skin and endocrine toxicities, which require careful attention and effective management in clinical settings.

Notably, this review differs from general discussions on tumor immunotherapy by highlighting the unique association between UV-induced mutations and Immune Evasion within the context of cSCC and the Tumor Microenvironment. In addition, the therapeutic advantages of immune checkpoint inhibitors in this disease are clarified. This focused investigation addresses a critical gap in understanding the specific mechanisms of immunotherapy for cSCC, thereby offering more precise theoretical support for clinical application.

To further improve the effectiveness of immunotherapy, ongoing research is actively investigating novel targets, combination treatment strategies, and potential biomarkers. Future research should place greater emphasis on regulating the immunosuppressive microenvironment, with particular focus on disrupting its protective role in tumor progression and enhancing the immune system’s capacity to eliminate tumor cells. Priority should also be given to investigating the application of bispecific antibodies in combination with immune checkpoint inhibitors in cSCC, while considering the influence of gender differences on treatment selection to further promote the development of personalized therapies for cSCC.

Optimizing combination immunotherapy represents another critical direction for future studies. By integrating immune checkpoint inhibitors with chemotherapy, radiotherapy, targeted therapy, and other treatment strategies, tumors can be targeted through multiple mechanisms, thereby improving overall therapeutic outcomes. In addition, the development of personalized treatment strategies remains a key focus of future research. By tailoring precise therapeutic approaches to individual patient characteristics and tumor-specific features, treatment efficacy can be maximized while minimizing adverse effects.

The integration of tumor metabolism and immunotherapy has also emerged as a prominent area of investigation. Clarifying how tumor metabolism affects immune responses, and how its modulation can enhance the effectiveness of immunotherapy, is expected to provide new insights for the treatment of cSCC. At the same time, the application of advanced technologies such as mass cytometry, single-cell transcriptomics, and epigenetics will enable a deeper understanding of the complex interactions between the Tumor Microenvironment and immune responses, thereby offering scientific evidence and novel strategies for optimizing immunotherapeutic approaches. By fostering multidisciplinary collaboration and innovation, ongoing progress in precision and personalized treatment for cSCC is expected to offer patients more effective therapeutic options and renewed hope, ultimately leading to improved prognosis and enhanced quality of life for those affected by cSCC.
